# MicroRNA16 regulates glioma cell proliferation, apoptosis and invasion by targeting Wip1-ATM-p53 feedback loop

**DOI:** 10.18632/oncotarget.18510

**Published:** 2017-06-16

**Authors:** Xiao-Hong Zhan, Qiu-Yan Xu, Rui Tian, Hong Yan, Min Zhang, Jing Wu, Wei Wang, Jie He

**Affiliations:** ^1^ School of Medicine, Shandong University, Jinan 250012, Shangdong Province, P.R. China; ^2^ Department of Pathology, Anhui Provincial Cancer Hospital; Anhui Provincial Hospital, Anhui Medical University, Hefei 230031, Anhui Province, P.R. China; ^3^ Department of Pathology, The Affiliated Central Hospital of Qingdao University, Qingdao 266000, Shandong Province, P.R. China; ^4^ Department of Medical Oncology, Anhui Provincial Hospital, Anhui Medical University, Hefei 230001, Anhui Province, P.R. China

**Keywords:** glioma, microRNA16, Wip1, ATM, p53

## Abstract

The present study aimed to investigate the role and underlying mechanisms of microRNA16 (miR-16) on proliferation, apoptosis and invasion of glioma cells. The cell models of miR-16 upregulation and Negative control group (NC group) were built. The cell functions of different groups were detected by colony formation assay, transwell chamber assay, proliferation, apoptosis and cycle experiments. The intracranial orthotopic transplantation animal models were built to different groups: miR-16 agomir group, miR-16 antagomir group and their NC group. The expressions of miR-16, Wip1, ATM and p53 were measured by qRT-PCR, western blot and immunohistochemistry. As a result, miR-16 overexpressed groups had lower cloning formation rate and proliferation rate, less invasive cells, higher early apoptosis rate than the control groups. G1 phase was significantly smaller compared miR-16 overexpressed groups with the control groups, and S phase significantly lesser. Cell growth was retardated. Differences were statistically significant (*P* <0.05). Compared with miR-16 overexpressed groups and NC groups, the Wip1 gene and protein expression were downregulated, while ATM and p53 genes, p-ATM and p-p53 proteins were upregulated. The differences were statistically significant (*P* <0.05). Taken together, our findings demonstrated that miR-16 suppressed glioma cell proliferation and invasion, promoted apoptosis and inhibited cell cycle by targeting Wip1-ATM-p53 signaling pathway.

## INTRODUCTION

Glioma accounts for 80% of all malignant primary brain tumors, with an annual incidence of approximately 6 per 100,000 in the USA [1]. It showed high mortality and recurrence rate, due to difficult to completely remove by operation, and not sensitive to radiotherapy and chemotherapy. Five year survival rate was only 20%˜30% [2]. Thus, it is critical to deeply explore the molecular mechanisms of glioma, providing clinical diagnostic markers and therapeutic targets.

MicroRNAs play important roles in the initiation and progression of tumors. As a member, microRNA16 (miR-16) has been found to be abnormally expressed in many kinds of tumors, presenting an interestingly two-sided phenomenon. In one hand, miR-16, although first reported in chronic lymphocytic leukemia (CLL) as a tumor-suppressor [3], was downregulated in many types of cancer, including human hepatoma [4], colorectal cancer [5], bladder cancer [6], prostate cancer [7], brain glioma [8] and different kinds of lymphoma or leukemia [9–13]. Restoring its level helped to enhance apoptosis and retard the cell-cycle of the cancer cells. In the other hand, miR-16 was also found to be upregulated in some types of cancer such as gastric cancer [14], pancreatic cancer [15], renal cell carcinoma [16] and ovarian carcinoma [17], indicating its potential role as an oncomiR. Moreover, Zhang et al. [18] have found that the transcripts of the Wip1 gene were specifically targeted by miR-16. Overexpression of miR-16 abolished the DNA damage-responsive Wip1 induction while inhibition of miR-16 markedly accelerated and enhanced the Wip1 induction. Oncogenic Wip1 phosphatase was inhibited by miR-16 in the DNA damage response and mammary tumorigenesis. At present, it has been found that there were at least 7 target proteins of Wip1, including p53, p38MAPK, ATM, CHK1, CHK2, MDM2 and UNG2 [19]. However, more detailed information about the function and molecular mechanisms of miR-16 in glioma still remains poorly understood.

Therefore, in the present study, miR-16 and related target genes Wip1, ATM and p53 were detected *in vivo* and *in vitro* experiments, and their effects on glioma and its possible mechanism were discussed.

## RESULTS

### Overexpression of miR-16 inhibited the proliferation and invasion of SHG44, U87 and U251 cells

**Figure 1:**
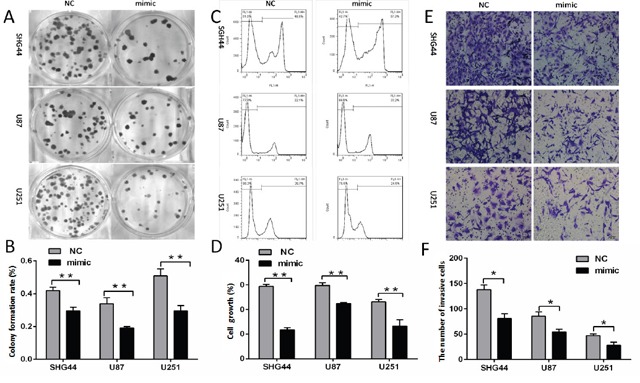
MiR-16 inhibited the proliferation and invasion of glioma cells **(A, B)** After transfection of miR-16 mimic and miR16 negative control, SHG44, U87 and U251 cells clone formation rates of miR-16 mimic groups were lower than those of the NC groups. The differences were statistically significant (*P* <0.01). **(C, D)** SHG44, U87, U251 cells proliferation assay results: the cell proliferation rates of miR-16 mimic groups were lower than those of NC groups, and the differences were statistically significant (*P* <0.01). **(E, F)** The average number of invasive cells per field of vision was significantly decreased from 137 to 74 in miR-16 mimic groups compared with the NC groups. The differences were statistically significant (*P* <0.05).

Clone formation and EdU proliferation experimental results suggested that clone formation rate and cell proliferation rate were lower in miR-16 mimic groups than those in NC groups (Figure 1A-1D). The results showed that overexpression of miR-16 inhibited the growth and proliferation of SHG44, U87 and U251 cells.

Cell invasion experiment showed that the average number of invasive cells per field of vision from 137 to 74 (Figure 1E and 1F) in miR-16 mimic groups compared with NC groups. It indicated that overexpression of miR-16 significantly inhibited the invasion of SHG44, U87 and U251 cells in matrix gel.

### Overexpression of miR-16 promoted tumor cell apoptosis and inhibited cell cycle progression

**Figure 2:**
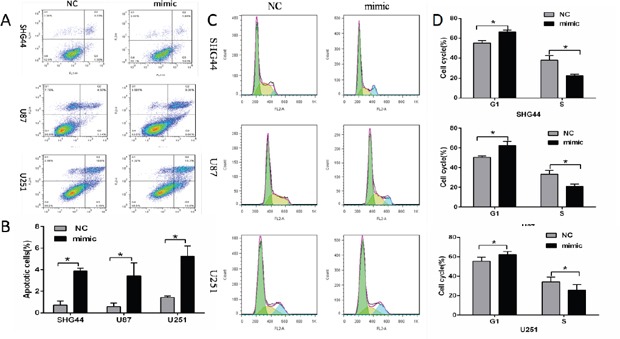
MiR-16 inhibited apoptosis and cell cycle of glioma cells **(A, B)** Early apoptosis rates of SHG44, U87 and U251 cells in miR-16 mimic groups were higher than those in NC groups, and the differences were statistically significant (*P* <0.05). **(C, D)** Compared with the NC groups, SHG44, U87 and U251 cells in miR-16 mimic groups were significantly more in G1 phase, and lesser in S phase. The differences were statistically significant (*P* <0.05).

Flow cytometry analysis showed that apoptosis rates of SHG44, U87 and U251 cells were higher in miR-16 mimic groups than those in NC groups (Figure 2A and 2B), suggesting that overexpression of miR16 promoted cell apoptosis.

Next, SHG44, U87 and U251 cells cycle were detected by the flow cytometry. Results showed that cells of G1 phases were significantly more in miR-16 mimic groups than those in NC groups, while cells of S phases were significantly lesser. Cell growth was restricted (Figure 2C and 2D). The role of miR-16 in inhibiting cell cycle was confirmed.

### Overexpression of miR-16 reduced glioma growth and invasion in an encephalic glioma nude mouse model

**Figure 3:**
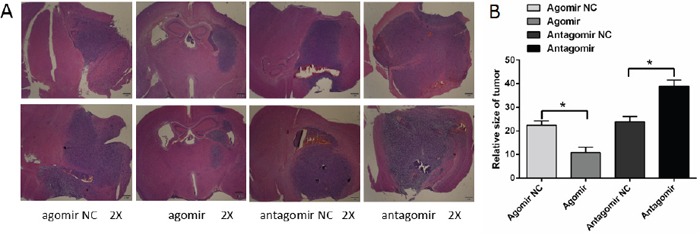
MiR-16 reduced glioma growth and invasion in an encephalic glioma nude mouse model **(A)** Microscopic image of the encephalic glioma (×2 magnification). The tumor size of miR-16 agomir group was smaller than that of NC group. The tumor size of miR-16 antagomir group was larger than that of NC group. **(B)** Statistical results of the tumor size in each group. The differences were statistically significant (*P* <0.05).

Tumor size of each group was calculated according to the following formula: tumor volume [mm^3^]= {(length [mm]) × (width [mm])}^2^/2 [20]. As shown in Figure 3, statistical results showed that: the tumor size of miR-16 agomir group was smaller than that of NC group, and the tumor size of miR-16 antagomir group was larger than that of NC group. The differences were statistically significant (*P* <0.05). This result was a direct description that overexpression of miR-16 could reduce glioma growth and invasion.

### Overexpression of miR-16 reduced the expression of Wip1 and increased the expressions of ATM and p53

**Figure 4:**
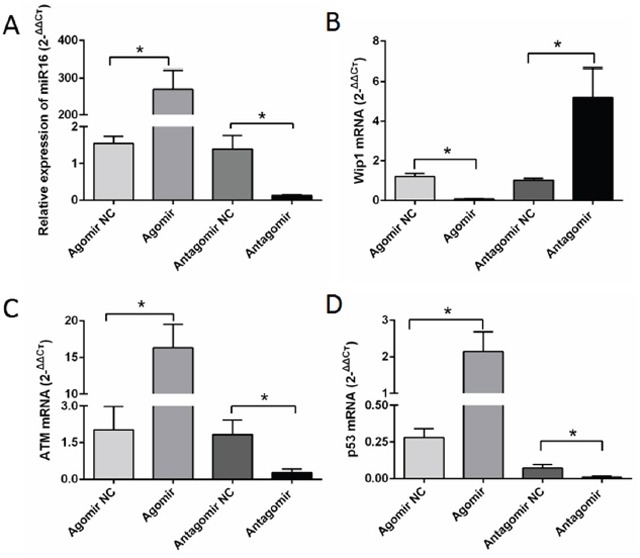
MiR-16 inhibited the expression of Wip1 and increased the expressions of ATM, p-ATM, p53 and p-p53 **(A)** qRT-PCR detected miR-16 expression of nude mice brain glioma. The expression of miR-16 was significantly higher in miR-16 agomir group than that in the NC group, and significantly lower in miR-16 antagomir group compared with NC group. The differences were statistically significant (*P* <0.05). **(B)** The expression of Wip1 was significantly lower in miR-16 agomir group than that in the NC group, and significantly higher in miR-16 antagomir group compared with NC group. The differences were statistically significant (*P* <0.05). **(C)** The expression of ATM was significantly higher in miR-16 agomir group than that in the NC group, and significantly lower in miR-16 antagomir group compared with NC group. The differences were statistically significant (*P* <0.05). **(D)** The expression of p53 was significantly higher in miR-16 agomir group than that in the NC group, and significantly lower in miR-16 antagomir group compared with NC group. The differences were statistically significant (*P* <0.05).

qRT-PCR was used to examine the expressions of miR-16, Wip1, ATM and p53 genes in SHG44, U87, U251 cells and brain glioma tissues of nude mice. The results showed that the expression of miR16 gene was higher in miR-16 agomir groups than that in NC groups, and lower in miR-16 antagomir groups than that in NC groups (Figure 4A). Besides, the expression of Wip1 gene was lower in miR-16 agomir groups than that in NC groups, while significantly higher in miR-16 antagomir groups than that in NC groups (Figure 4B). Conversely, the expressions of ATM and p53 genes were higher in miR-16 agomir groups than those in NC groups, while significantly decreased in miR-16 antagomir groups compared with NC groups (Figure 4C and 4D). The study confirmed that miR-16 inhibited the expression of Wip1 gene, while increased the expression of ATM and p53 genes.

**Figure 5:**
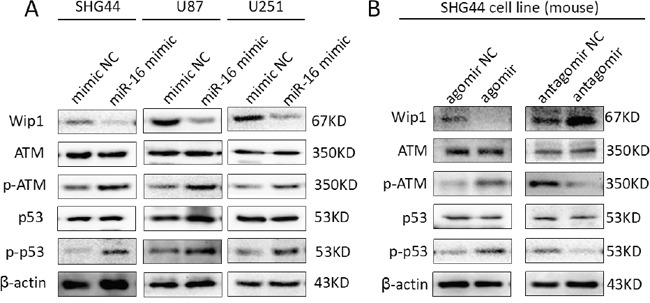
MiR-16 inhibited the protein expression of Wip1 and increased the protein expressions of ATM, p-ATM, p53 and p-p53 **(A)** Western blot results of SHG44, U87 and U251 cell lines showed that: the expression of Wip1 protein was lower in miR-16 mimic groups than that in NC groups. Conversely, expressions of p-ATM (s1981) and p-p53 (s15) proteins were higher compared miR-16 mimic groups with NC groups. **(B)** In brain glioma tissues of nude mice, it was lower expression of Wip1 protein and higher expressions of p-ATM (s1981) and p-p53 (s15) proteins in miR-16 agomir groups than that in NC groups, and higher expression of Wip1 protein and lower expressions of p-ATM (s1981) and p-p53 (s15) proteins in miR-16 antagomir groups than that in NC groups.

**Figure 6:**
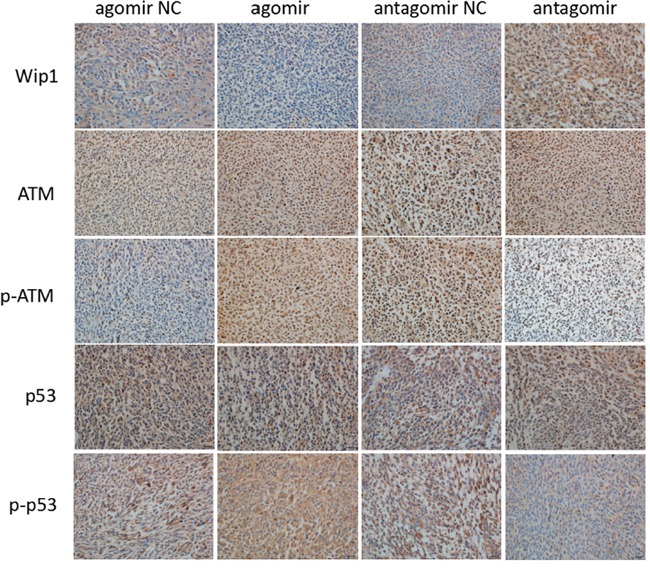
MiR-16 inhibited the protein expression of Wip1 and increased the protein expressions of ATM, p-ATM, p53 and p-p53 In brain glioma tissues of nude mice, the immunohistochemical results were lower expression of Wip1 protein and higher protein expressions of p-ATM and p-p53 in miR-16 agomir groups than those in NC groups, and higher expression of Wip1 protein and lower protein expressions of p-ATM and p-p53 in miR-16 antagomir groups than those in NC groups.

Also, the protein expressions of Wip1, p-ATM and p-p53 were detected by western blot and immunohistochemistry. The results showed it was lower expression of Wip1 protein and higher expression of p-ATM and p-p53 proteins compared miR-16 agomir groups with NC groups. Conversely, it was higher expression of Wip1 protein and lower expression of p-ATM and p-p53 proteins compared miR-16 antagomir groups with NC groups (Figures 5 and 6). It was similar to the result of qRT-PCR, indicating that miR-16 could also increase the protein expressions of p-ATM and p-p53 by inhibiting the protein expression of Wip1.

### MiR-16 directly targets Wip1 in glioma cell

**Figure 7:**
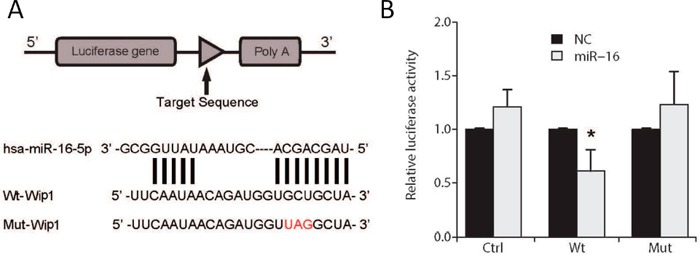
Luciferase assay in U87 cells **(A)** Schematic diagram of the Wip1 3’-UTR reporter construct. **(B)** Luciferase activity was measured as relative activity to the corresponding normal control (NC) (mock, assigned as value “1”). Values denote the mean ± SEM of three independent assays.

To further support the observation that Wip1 is the direct target of miR-16, luciferase assays were performed using glioma cells transfected with Wip1 reporter constructs with or without miR16 (Figure 7A). As expected, luciferase activity in glioma cells was reduced by co-transfection of the Wip1 construct and miR16, while mutation of Wip1's 3’-UTR miR-16 binding sites abrogated reduction of luciferase activity by miR-16 (Figure 7B). The data indicates that miR-16 directly targets Wip1 in glioma cells.

## DISCUSSION

MiR-16 was first discovered by Calin et al. [3] in CLL, which was proved to play an important role in tumorigenesis as a tumor suppressor gene. Thereafter, in the field of cancer, some studies have found miR-16 acts as a role of suppressor gene [3–13], while other studies have demonstrated its role as an oncomiR [14–17]. As for this seemingly contradictory issue, what are the exact role of miR-16 in glioma and its underlying mechanisms attracts our study interest.

In the present study, we first predicted that the 3'UTR of wild-type p53-induced phosphatase 1 (Wip1) and miR-16 can bind complementarily by Targetscan and microRNA network tools, suggesting that Wip1 may be a direct target gene of miR-16. The prediction was then confirmed by Luciferase assay. Wip1 belongs to the serine/threonine protein phosphatase, which is a member of the protein phosphatase 2C (PP2C) family, located in the human chromosome 17q22/q24, by protein phosphatase magnesiumdependent 1 delta (PPMlD) gene encoding [21]. As a member of the PP2C and p53 target gene family, once Wip1 is activated by p53, it will directly cause the downstream target protein dephosphorylation and inactivation. Till now, it has been found that there are at least 7 target proteins of Wip1, including p53, p38MAPK, ATM, CHK1, CHK2, MDM2 and UNG2 [19]. Besides, recent studies have demonstrated that H2AX also serves as a target of Wip1 [22–27].

ATM kinase is a key tumor suppressor. Its activation can phosphorylate a variety of different target proteins inducing cell cycle arrest, DNA repair and apoptosis [28]. Human HDM2 homolog (MDM2) is an E3 ubiquitin ligase that can specifically degrade p53. ATM can be directly combined with p53 and MDM2, leading to phosphorylation of p53 and MDM2, blocking the degradation of MDM2 on p53, maintaining the level and stability of p53 directly and indirectly [29]. ATM can also phosphorylate SQ domain of CHK2 to phosphorylate ser15, 20 sites of p53, which activates its downstream p21 gene. P21 protein is a cyclin-dependent kinase inhibitor (CDI), which can inhibit CyclinE/Cdk2, causing G1 block and resulting in G1/S cell cycle arrest [30, 31]. The results of our experiments showed that the expression levels of ATM and p53 gene and p-ATM and p-p53 protein were high when Wip1 was low expression. In contrast, the expression levels of ATM and p53 gene and p-ATM and p-p53 protein were significantly decreased when Wip1 was high expression. Thus, Wip1 was proved to be an inhibitor of ATM kinase, which could inactivate ATM by dephosphorylation, to reduce the activity and expression of p53.

As the main tumor suppressor gene, p53 has the role of blocking cell cycle, inducing cell apoptosis and promoting DNA repair, so as to avoid accumulation of DNA damage and maintain the stability of the genetic and prevent cell transformation. It is of great significance in preventing the occurrence of tumors [32]. Wip1 can directly phosphorylate Ser15 site of p53 to make it inactive. It also can dephosphorylate Thr180 site of p38MAPK, inhibit p38MAPK activity and then block the phosphorylation of Ser33 and Ser46 of p53, which is the downstream target gene of p38MAPK. Wip1 can make p53 dephosphorylated and inactivated by dephosphorylating the Ser345 sites of CHK1 and CHK2, leading to the occurrence of tumor. By dephosphorylation of MDM2 and making its stability, Wip1 can enhance the interaction between MDM2 and p53, to promote the degradation of p53 protein [33–35]. Our study showed that Wip1 can suppress p53 gene and p-p53 protein. When overexpression of Wip1, p53 gene and p-p53 protein were both decreased.

In this study, our findings showed that miR-16 can significantly inhibit cell proliferation and invasion, promote cell apoptosis and block cell cycle progression. qRT-PCR, western blot and immunohistochemistry showed that the expressions of Wip1 gene and protein decreased obviously in the group of miR-16 overexpression compared with those in the group of miR-16 knockdown. Conversely, ATM and p53 gene and p-ATM and p-p53 proteins increased overexpression in the groups of miR-16 overexpression compared with those in the groups of miR-16 knockdown. This result indicates that Wip1 is the target gene of miR-16, and miR-16 can inhibit the expression of Wip1 [18, 36]. While Wip1 is ATM and p53 inhibitor, when the over expression of miR-16, both ATM and p53 increase expression because of losing the inhibition of Wip1, then perform the function of blocking the cell cycle and inhibiting cell proliferation and invasion. Therefore, miR-16 can play an important role in the inhibition of tumor by targeting the Wip1-ATM-p53 signaling loop in glioma.

Further, we studied the effects of miR-16 regulating Wip1-ATM-p53 signaling pathway on glioma by *in vivo* and *in vitro* experiments. The results confirmed that miR-16 reduced glioma growth and invasion. Because the therapeutic challenge of glioma due to the lack of molecular targets, we predict that miR-16 might provide new insights into the development of therapeutic strategies against glioma.

However, some limitations should be noted. First, due to lack of clinical tissue and corresponding follow-up data, the prognostic significance cannot be observed in this study. Second, when we explore the underlying molecular mechanism, some of deeper methods are not be used, like rescue experiment. Third, our preliminary findings only showed that miR-16 regulated Wip1-ATM-P53 signaling loop to influence the cell proliferation, invasion, apoptosis and cell cycle of glioma, but whether miR-16 is Wip1 or p53 dependent wasn't directly explained in this study. All these limitations will be solved in our further experiments.

In conclusion, our findings demonstrated that miR-16 suppressed glioma cells proliferation and invasion, promoted apoptosis and inhibited cell cycle by targeting Wip1-ATM-p53 signaling pathway.

## MATERIALS AND METHODS

### Cell lines, animals and reagents

Human glioma cell lines SHG44, U87 and U251 were purchased from the Chinese typical culture preservation center. Nude mice, 4-5 weeks of age, male, were purchased from Suzhou Industrial Park Aier Matt Technology Co., Ltd. (Certificate No. 201505453). DMEM culture medium and fetal bovine serum were purchased from American Gibco Corporation. 0.25% pancreatin, bovine serum albumin (BSA), RNase was purchased from Shanghai Beyotime Institute of Biotechnology. Lipofecamine 2000 was purchased from the United States Invitrogen company. MiR-16 mimic, agomir and antagomir transfection kit were purchased from Guangzhou RiboBio Co., Ltd.. Matrigel was purchased from American Becton Dickinson Corporation. Wip1 (ab31070, rabbit polyclonal antibody), ATM (ab78, mouse monoclonal antibody), p53 (ab28, mouse monoclonal antibody), p-ATM (ab36810, mouse monoclona antibody) and p-P53 (ab1431, rabbit polyclonal antibody) were purchased from abcam UK Ltd.. Goat anti-rabbit antibody, goat anti-mouse antibody and β-actin were purchased from Beijing Zhoushan Golden Bridge Biotechnology Co., Ltd.. High-Capacity cDNA Reverse Transcription Kits, TaqMan Universal Master Mix II, microRNA16 probe primer and internal reference U6 probe primer were purchased from American ABI company, as also as Wip1, ATM, p53 and β-actin probe primers.

### Cell culture and transfection

Human glioma cell lines SHG44, U87, and U251 were cultured in high glucose DMEM medium supplemented with 10% fetal bovine serum, and placed at 37°C in 5% CO_2_ incubator. Routine passage was performed. When in the logarithmic phase, the cells were digested, collected and inoculated in 6-well plate at 1×10^5^ cells per well. When the cell fusion rate reached about 30%, Lipofecamine 2000 was used to transfect cells with miR-16 mimic and non-targeting negative control (NC), or miR-16 agomir, antagomir and their negative control were transfected directly. After 48 hours of transfection, the cells were collected for the after experiments.

### Clone formation experiment

Transfected with miR-16 mimic and non-targeting negative control, cells were collected, counted after 48 hours, and seeded at low density (200 cells/well) in a 6-well plate. After 4-5 days, medium was exchanged. Culture was terminated about 10-12 days. Cells were fixed in 4% formalin, and dyed by 0.1% crystal violet. The number of clones with more 50 cells was counted under the microscope. The rate of clone formation (%) = the number of clones/actual inoculation cell number × 100%. Each group had 3 duplicate wells and 3 times repeated.

### Cell invasion experiment

The matrigel was diluted at the rate of 1: 8 by cold DMEM, then added to chambers (100 μl/chamber), and placed 2 hours at 37°C. The top chamber was added with 100 μl cell suspension (1 ×10^6^ cells/ml with no serum medium), and the bottom chamber with 600 μl complete culture medium containing 10% FBS. After 24 h incubation box, removed the chamber, and wiped off the inner surface cells with cotton swab, then fixed in 4% formalin, finally dyed by 0.1% crystal violet. We counted randomly the number of cells in 8 fields of view under 200 × microscope. MiR-16 mimic group and NC group were both repeated 3 times.

### Proliferation, apoptosis and cycle experiments

After digested with pancreatin without EDTA, cells were collected and washed two times with pre-cold PBS, and then dealt with according to the instruction of Annexin V-488/PI Apoptosis Kit, EdU Proliferation Kit, and cycle experiment. Finally, cells were detected by flow cytometry.

### Intracranial orthotopic transplantation tumor in nude mice [37]

Animal experiments were practiced according to the ethics committee of Medical University of Anhui [No. (2013) 44]. Nude mice were divided into miR-16-agomir group and NC group, miR-16-antagomir group and NC group. There were five nude mice in each group. After transfection, SHG44 cells were made into PBS cell suspension, counted and adjusted to the concentration of 2 × 10^6^ cells/μl. After intraperitoneal anesthesia, the nude mice were fixed on the single arm digital stereotaxis instrument (ShangHai Biowill Co., Ltd.) Disinfected the scalp and cut it open for exposure of surgical field (1 mm before the anterior fontanelle, 3 mm besides sagittal suture). Then the skull drill (ShangHai Biowill Co., Ltd.) was use to drill the skull, and the micro injector was used to inject 5 μl cell suspension into the brain of nude mice (the depth of needle insertion was 3.5 mm). We pumped needle slowly, use bone wax to seal the drill, bonded scalp by medical adhesive and disinfected it again. After the operation, nude mice were weight and observed every day. When occurred cachexia, nude mice were breathed CO_2_ to euthanasia. The brains were completely removed. Parts of fresh brain tumor tissue were used to RT-PCR and Western-Blot experiments. All the remaining brain tissues were fixed in 10% neutral formalin, embedded by paraffin, then with conventional HE staining and immunohistochemical staining.

### qRT-PCR experiment

The total RNA of brain tumors were extracted with total RNA kit II, and the cDNA was synthesized using High-Capacity cDNA Reverse Transcription Kit. MiR-16, Wip1, ATM and p53 were examined by TaqMan Universal Master MixIIkit, and β-actin as the reference gene for correction. ABI 7500 real time fluorescence PCR instrument (American ABI company) reaction condition: denaturation (95°C, 15s), annealing/extension (60°C, 60s), the number of cycles: 45 cycles. MiR-16 primer sequences: UAGCAGCACGUAAAUAUUGGCG, Wip1 primer sequences: TGGAAGAAACTGGCGGAATGGCCAA, ATM primer sequences: GCTACAGAACGAAAGAAAGAAGTTG, p53 primer sequences: GCTCACTCCAGCCACCTGAAGTCCA.

### Western blot experiment

Total protein was extracted using RIPA lysis solution (protease inhibitor). Protein concentration was examined by BCA method. After routine denaturation, proteins were separated by 10% SDS-PAGE and transferred to PVDF films by the wet transfer method. 5% skim milk was used to block antibodies for 2 hours at room temperature. Add the first antibody and stay overnight at 4°C. After washing the membrane by TBST, add the second antibody and keep 2 hours at room temperature. After the membrane was washed, the protein expressions of Wip1, ATM, p-ATM (phospho s1981), P53 and p-P53 (phospho s15) were detected by BCL chemiluminescence method, and β-actin as the reference protein for correction. Densitometric analysis of protein bands was performed via using Image J software. Three times were repeated. The first antibody dilution concentration was: Wip1 (1: 100), p53 (1: 200), p-P53 (1: 100), ATM (1: 100), p-ATM (1: 100). The second antibody dilution concentration was 1: 1000.

### Immunohistochemical staining

Immunohistochemical staining of Wip1, ATM, p-ATM, p53 and p-p53 proteins was performed by a fully automated immunohistochemistry (Roche Ventana). All procedures were performed in accordance with the instruments and reagents. Antibodies dilution was all 1: 500. The antigen retrieval method was citric acid thermal repair. Finally DAB staining was suitable. Substitution of PBS for the primary antibody served as negative control. Staining results were observed under microscope: Wip1, p53 and p-p53 proteins were localized in the nucleus and cytoplasm, while ATM and p-ATM located in the nucleus. The positive staining was light yellow, yellow or brown. Ten randomly chosen visual fields were observed on every section under microscope. Semi quantitative analysis of immunohistochemical method was performed [38]. Calculate the percentage of positive stained cells: less than 5% for 0 point; 6% to 25% for 1 point: 26% to 50% for 2 points: 51% to 75% 3 for points; >75% for 4 points. Strength grading of staining was negative for 0 point, light yellow for 1 point; yellow or dark yellow 2 points, brown or dark brown for 3 points. Both score multiplied was the final score of the immunohistochemistry.

### Vector construction

MiR-16 gene was amplified by PCR from genomic DNA isolated from human brain tissue and cloned into vector pcDNA3.1 (Promega, Madison, WI, USA). A 3’-untranslated region (UTR) luciferase reporter vector was constructed by ligating a fragment of the Wip1 3’-UTR encompassing the miR16 binding sequence into the pcDNA3.1-luc vector (Promega).

### Luciferase assay

U87 cells were plated in 24-well plates for 24 hours, and then co-transfected with miR-16 or pcDNA3.1-luc vector containing wild-type or mutant 3’UTR using lipofectamine 2000. Luciferase assays were performed 48 hours after transfection using the Luciferase Reporter Assay System (Promega) according to the manufacturer's instructions.

### Statistical analysis

SPSS 22.0 software was used to deal experimental data, two independent samples *t* test was used to analyze the discrimination of groups. A *P*-value of less than 0.05 was considered to be statistically.

## References

[1] Ostrom QT, Gittleman H, Fulop J, Liu M, Blanda R, Kromer C, Wolinsky Y, Kruchko C, Barnholtz-Sloan JS (2015). CBTRUS statistical report: primary brain and central nervous system tumors diagnosed in the united states in 2008-2012. Neuro Oncol.

[2] Louis DN, Ohgaki H, Wiestler OD, Cavenee WK, Burger PC, Jouvet A, Scheithauer BW, Kleihues P (2007). The 2007 WHO classification of tumours of the central nervous system. Acta Neuropathol.

[3] Calin GA, Dumitru CD, Shimizu M, Bichi R, Zupo S, Noch E, Aldler H, Rattan S, Keating M, Rai K, Rassenti L, Kipps T, Negrini M (2002). Frequent deletions and down-regulation of micro- RNA genes miR15 and miR16 at 13q14 in chronic lymphocytic leukemia. Proc Natl Acad Sci U S A.

[4] Agra Andrieu N, Motiño O, Mayoral R, Llorente Izquierdo C, Fernández-Alvarez A, Boscá L, Casado M, Martín-Sanz P (2012). Cyclooxygenase-2 is a target of microRNA-16 in human hepatoma cells. PLoS One.

[5] Qian J, Jiang B, Li M, Chen J, Fang M (2013). Prognostic significance of microRNA-16 expression in human colorectal cancer. World J Surg.

[6] Jiang QQ, Liu B, Yuan T (2013). MicroRNA-16 inhibits bladder cancer proliferation by targeting Cyclin D1. Asian Pac J Cancer Prev.

[7] Bonci D, Coppola V, Musumeci M, Addario A, Giuffrida R, Memeo L, D'Urso L, Pagliuca A, Biffoni M, Labbaye C, Bartucci M, Muto G, Peschle C, De Maria R (2008). The miR-15a-miR-16-1 cluster controls prostate cancer by targeting multiple oncogenic activities. Nat Med.

[8] Yang TQ, Lu XJ, Wu TF, Ding DD, Zhao ZH, Chen GL, Xie XS, Li B, Wei YX, Guo LC, Zhang Y, Huang YL, Zhou YX, Du ZW (2014). MicroRNA-16 inhibits glioma cell growth and invasion through suppression of BCL2 and the nuclear factor-κB1/MMP9 signaling pathway. Cancer Sci.

[9] Dejean E, Renalier MH, Foisseau M, Agirre X, Joseph N, de Paiva GR, Al Saati T, Soulier J, Desjobert C, Lamant L, Prósper F, Felsher DW, Cavaillé J (2011). Hypoxia-microRNA-16 downregulation induces VEGF expression in anaplastic lymphoma kinase (ALK)-positive anaplastic large-cell lymphomas. Leukemia.

[10] Liu J, Chen G, Feng L, Zhang W, Pelicano H, Wang F, Ogasawara MA, Lu W, Amin HM, Croce CM, Keating MJ, Huang P (2014). Loss of p53 and altered miR15-a/16-1→MCL-1 pathway in CLL: insights from TCL1-Tg: p53−/− mouse model and primary human leukemia cells. Leukemia.

[11] Fabbri M, Bottoni A, Shimizu M, Spizzo R, Nicoloso MS, Rossi S, Barbarotto E, Cimmino A, Adair B, Wojcik SE, Valeri N, Calore F, Sampath D (2011). Association of a microRNA/TP53 feedback circuitry with pathogenesis and outcome of B-cell chronic lymphocytic leukemia. JAMA.

[12] Teshima K, Nara M, Watanabe A, Ito M, Ikeda S, Hatano Y, Oshima K, Seto M, Sawada K, Tagawa H (2014). Dysregulation of BMI1 and microRNA-16 collaborate to enhance an anti-apoptotic potential in the side population of refractory mantle cell lymphoma. Oncogene.

[13] Zhang X, Chen X, Lin J, Lwin T, Wright G, Moscinski LC, Dalton WS, Seto E, Wright K, Sotomayor E, Tao J (2012). Myc represses miR-15a/miR-16-1 expression through recruitment of HDAC3 in mantle cell and other non-Hodgkin B-cell lymphomas. Oncogene.

[14] Wang H, Wang L, Wu Z, Sun R, Jin H, Ma J, Liu L, Ling R, Yi J, Wang L, Bian J, Chen J, Li N (2014). Three dysregulated microRNAs in serum as novel biomarkers for gastric cancer screening. Med Oncol.

[15] Tian Y, Xue Y, Ruan G, Cheng K, Tian J, Qiu Q, Xiao M, Li H, Yang H, Wang L (2015). Interaction of serum microRNAs and serum folate with the susceptibility to pancreatic cancer. Pancreas.

[16] Chen D, Li Y, Yu Z, Su Z, Yu W, Li Y, Yang S, Gui Y, Ni L, Lai Y (2015). Upregulated microRNA-16 as an oncogene in renal cell carcinoma. Mol Med Rep.

[17] Miles GD, Seiler M, Rodriguez L, Rajagopal G, Bhanot G (2012). Identifying microRNA/mRNA dysregulations in ovarian cancer. BMC Res Notes.

[18] Zhang X, Wan G, Mlotshwa S, Vance V, Berger FG, Chen H, Lu X (2010). Oncogenic Wip1 phosphatase is inhibited by miR-16 in the DNA damage signaling pathway. Cancer Res.

[19] Emelyanov A, Bulavin DV (2015). Wip1 phosphatase in breast cancer. Oncogene.

[20] Naito S, von Eschenbach AC, Giavazzi R, Fidler IJ (1986). Growth and metastasis of tumor cells isolated from a human renal cell carcinoma implanted into different organs of nude mice. Cancer Res.

[21] Buss MC, Remke M, Lee J, Gandhi K, Schniederjan MJ, Kool M, Northcott PA, Pfister SM, Taylor MD, Castellino RC (2015). The WIP1 oncogene promotes progression and invasion of aggressive medulloblastoma varians. Oncogene.

[22] Macůrek L, Lindqvist A, Voets O, Kool J, Vos HR, Medema RH (2010). Wip1 phosphatase is associated with chromatin and dephosphorylates gammaH2AX to promote checkpoint inhibition. Oncogene.

[23] Moon SH, Lin L, Zhang X, Nguyen TA, Darlington Y, Waldman AS, Lu X, Donehower LA (2010). Wild-type p53-induced phosphatase 1 dephosphorylates histone variant gamma-H2AX and suppresses DNA double strand break repair. J Biol Chem.

[24] Cha H, Lowe JM, Li H, Lee JS, Belova GI, Bulavin DV, Fornace AJ (2010). Wip1 directly dephosphorylates gamma-H2AX and attenuates the DNA damage response. Cancer Res.

[25] Sakai H, Fujigaki H, Mazur SJ, Appella E (2014). Wild-type p53-induced phosphatase 1 (Wip1) forestalls cellular premature senescence at physiological oxygen levels by regulating DNA damage response signaling during DNA replication. Cell Cycle.

[26] Mirzayans R, Andrais B, Scott A, Wang YW, Weiss RH, Murray D (2015). Spontaneous γH2AX foci in human solid tumor-derived cell lines in relation to p21waf1 andwip1 expression. Int J Mol Sci.

[27] He ZY, Wang WY, Hu WY, Yang L, Li Y, Zhang WY, Yang YS, Liu SC, Zhang FL, Mei R, Xing D, Xiao ZC, Zhang M (2016). Gamma-H2AX upregulation caused by Wip1 deficiency increases depression-related cellular senescence in hippocampus. Sci Rep.

[28] Weber AM, Ryan AJ (2015). ATM and ATR as therapeutic targets in cancer. Pharmacol Ther.

[29] Inoue K, Fry EA, Frazier DP (2016). Transcription factors that interact with P53 and Mdm2. Int J Cancer.

[30] Malbert-Colas L, Ponnuswamy A, Olivares-Illana V, Tournillon AS, Naski N, Fåhraeus R (2014). HDMX folds the nascent p53 Mrna following activation by the ATM kinase. Mol Cell.

[31] Brazina J, Svadlenka J, Macurek L, Andera L, Hodny Z, Bartek J, Hanzlikova H (2015). DNA damage-induced regμlatory interplay between DAXX, p53, ATM kinase and Wip1 phosphatase. Cell Cycle.

[32] Marcel V, Catez F, Diaz JJ (2015). P53, a translational regulator: contribution to its tumor-suppressor activity. Oncogene.

[33] Goloudina AR, Kochetkova EY, Pospelova TV, Demidov ON (2016). Wip1 phosphatase: between P53 and MAPK kinases pathways. Oncotarget.

[34] Yi W, Hu X, Chen Z, Liu L, Tian Y, Chen H, Cong YS, Yang F, Zhang L, Rudolph KL, Zhang Z, Zhao Y, Ju Z (2015). Phosphatase Wip1 controls antigen-independent B-cell development in a p53-dependent manner. Blood.

[35] Esfandiari A, Hawthorne TA, Nakjang S, Lunec J (2016). Chemical inhibition of wild-type p53-induced phosphatase 1 (WIP1/PPM1D) by GSK2830371 potentiates the sensitivity to MDM2 inhibitors in a p53-dependent manner. Mol Cancer Ther.

[36] Rahman M, Lovat F, Romano G, Calore F, Acunzo M, Bell EH, Nana-Sinkam P (2014). MiR-15b/16-2 regulates factors that promote p53 phosphorylation and augments the DNA damage response following radiation in the lung. J Biol Chem.

[37] Wang H, Sun T, Hu J, Zhang R, Rao Y, Wang S, Chen R, McLendon RE, Friedman AH, Keir ST, Bigner DD, Li QJ, Wang H, Wang XF (2014). miR-33a promotes glioma-initiating cell self-renewal via PKA and NOTCH pathways. J Clin Invest.

[38] Takenoue T, Kitayama J, Takei Y, Umetani N, Matsuda K, Nita ME, Hatano K, Tsuruo T, Nagawa H (2000). Characterization of dihydropyrimidine dehydrogenase on immunohistochemistry in colon carcinoma, and correlation between immunohistochemical score and protein level or messenger RNA expression. Ann Oncol.

